# Risk of Fracture with Thiazolidinediones: Disease or Drugs?

**DOI:** 10.1007/s00223-012-9591-8

**Published:** 2012-04-10

**Authors:** Marloes T. Bazelier, Peter Vestergaard, Arlene M. Gallagher, Tjeerd-Pieter van Staa, Cyrus Cooper, Hubert G. M. Leufkens, Frank de Vries

**Affiliations:** 1 Utrecht Institute for Pharmaceutical Sciences, Utrecht University, Utrecht, The Netherlands; 2 The Osteoporosis Clinic, Aarhus University Hospital, Aarhus, Denmark; 3 General Practice Research Database, Medicines and Healthcare Products Regulatory Agency, London, UK; 4 MRC Lifecourse Epidemiology Unit, University of Southampton, Southampton, UK; 5 Institute of Musculoskeletal Sciences, University of Oxford, Oxford, UK; 6 Department of Clinical Pharmacy and Toxicology, Maastricht University Medical Centre, Maastricht, The Netherlands; 7 Department of Pharmacoepidemiology and Clinical Pharmacology, Utrecht Institute for Pharmaceutical Sciences, Utrecht University, Universiteitsweg 99, 3584 CG Utrecht, The Netherlands

**Keywords:** Thiazolidinedione, Type 2 diabetes mellitus, Fracture risk, Osteoporosis

## Abstract

The use of thiazolidinediones (TZDs) has been associated with an increased fracture risk. In addition, type 2 diabetes mellitus (T2DM) has been linked with fracture. We evaluated to what extent the association between TZD use and fracture risk is related to the drug or to the underlying disease. We conducted a population-based cohort study using the Danish National Health Registers (1996–2007), which link pharmacy data to the national hospital registry. Oral antidiabetic users (*n* = 180,049) were matched 1:3 by year of birth and sex to nonusers. Cox proportional hazards models were used to estimate hazard ratios (HRs) of fracture. Time-dependent adjustments were made for age, comorbidity, and drug use. We created a proxy indicator for the severity of disease. The first stage was defined as current use of either a biguanide or a sulfonyluerum, the second stage as current use of a biguanide and a sulfonyluerum at the same time, the third stage as patients using TZDs, and the fourth stage as patients using insulin. The risk of osteoporotic fracture was increased 1.3-fold for stages 3 and 4 compared with controls. Risk with current TZD use (stage 3 HR = 1.27, 95 % CI 1.06–1.52) and risk with current use of insulin (stage 4 HR = 1.25, 95 % CI 1.20–1.31) were similar. In the first (HR = 1.15, 95 % CI 1.13–1.18) and second (HR = 1.00, 95 % CI 0.96–1.04) stages risks were lower. Risk of osteoporotic fracture was similar for TZD users and insulin users. When studying fracture risk with TZDs, the underlying T2DM should be taken into account.

Thiazolidinediones (TZDs) improve insulin sensitivity through activation of the nuclear receptor peroxisome proliferator-activated receptor gamma (PPARγ) [1]. Until recently, both rosiglitazone and pioglitazone were frequently used in the management of type 2 diabetes mellitus (T2DM). An association of rosiglitazone with risk of cardiovascular outcomes has caused withdrawal of the drug in Europe and restricted access in the United States [2]. Pioglitazone is still used in the management of later stages of T2DM and has benefits in patients with a recent acute myocardial infarction [3].

Various studies have found that TZD use leads to decreased bone mineral density (BMD) and an elevated risk of fracture [4–10]. TZDs affect the differentiation of mesenchymal stem cells, leading to increased adipogenesis at the expense of the formation of osteoblasts [4, 5]. The use of TZDs in rodents has been linked with adverse skeletal effects [6, 7]. In humans, TZD use has also been associated with adverse skeletal outcomes, at least in women with T2DM: women who were exposed to TZDs for 3–4 months had significantly reduced BMD at the lumbar spine and hip in two randomized controlled trials [8, 9]. Moreover, a meta-analysis from ten randomized controlled trials showed that rosiglitazone and pioglitazone significantly increased the risk of fractures [odds ratio (OR) = 1.45, 95 % confidence interval (CI) 1.18–1.79] [10]. In observational studies, the relationship between TZD use and fracture risk has also been reported [11–14].

Not only the use of TZDs but also underlying T2DM has been associated with fracture [15, 16]. Among the potential mechanisms are direct effects of high glucose levels on bone turnover [17], increased urine calcium loss [18], changes in vitamin D metabolism [19], and alterations in glycosylation of collagen caused by hyperglycemia [20]. In addition, complications of diabetes such as renal failure, neuropathy, and micro- and macro-angopathy may contribute to fracture risk [21–24].

At present, it is unclear to what extent the association between TZD use and fracture risk is related to the drug or to the underlying disease. The aim of this study was to estimate risk of fracture in diabetic patients compared with controls, stratified by the use of TZDs and by disease severity.

## Methods

### Data Source

In Denmark, separate registers of computerized medical records on all contacts to hospitals and on the use of drugs can be linked for the entire population (approximately 5.5 million inhabitants). The Ministry of the interior keeps records of all inhabitants, including their migrations and dates of birth and death. Information on hospital admissions comes from the National Hospital Discharge Register [25], which covers all inpatient contacts from 1977 onwards and from 1995 also all outpatient visits to hospitals, outpatient clinics, and emergency rooms. Upon discharge, the physician codes the reason for the contact using the ICD system. The register has an almost 100 % capture of contacts, and the validity of registrations is high [26]. The Danish Medicines Agency keeps a nationwide register of all prescription drugs sold at pharmacies throughout the country from 1996 onward, the National Pharmacological Database (www.dkma.dk). All prescriptions are registered with ATC code, dosage, and date. As all sales are registered to the individual who redeems the prescription, the capture and validity are high. All registers can be linked through the use of a person-specific code (the civil person number) given to all inhabitants and can be used for all of the registrations mentioned.

### Study Population

The exposed cohort consisted of all patients (aged 18+) with at least one prescription of oral antidiabetic (OAD) medication between 1996 and 2007. Each OAD drug user was matched to up to three patients by exact year of birth and sex. Controls did not have an antidiabetic (OAD or insulin) prescription any time during follow-up. The date of the first OAD prescription defined the index date, and controls were assigned the same index date as their matched OAD drug user. All participants were then followed from their index date to the end of data collection, emigration, or the patient’s death, whichever came first.

### Exposure

The total period of follow-up for each patient was divided into periods of current exposure and past exposure, with patients moving between current and past use. Each period of current exposure started with an OAD or insulin prescription and ended 3 months after the expected duration of antidiabetic (AD) therapy or on the date that a new AD drug was prescribed within this period. The expected duration of OAD therapy was defined as the median expected duration of treatment, based on all OAD prescriptions. For insulin treatment, the median time between two insulin injections (based on all insulin prescriptions) was taken as the expected duration of use.

At the start of each interval, patients were classified as current users of AD medication if they had an AD prescription on that start date or in the 3 months before. Current user status was determined for five different groups of AD medication: biguanides, sulfonyluerum, TZDs, insulin, and other ADs [including dipeptidyl peptidase-4 (DPP-4) inhibitors, glinides, glucagon-like peptide-1 (GLP-1) analogs and α-glucosidase inhibitors]. For controls, the total period of follow-up was divided into periods of 6 months.

We created a proxy indicator for the severity of disease in diabetic patients. The first stage of disease was defined as current use of either a biguanide or a sulfonyluerum, the second stage comprised current users of a biguanide and a sulfonyluerum at the same time, the third stage was assigned to patients using TZDs (regardless of comedication), and the fourth stage was assigned to patients using insulin (regardless of comedication and not exposed to TZDs).

### Study Outcomes

Patients were followed up for the occurrence of a first fracture. Types of fracture were classified according to the International Classification of Diseases (ICD-10) categories. These included skull (S02), neck (S12), ribs (S22), pelvis (S32), shoulder (S42), forearm (S52), hand (S62), hip/femur (S72), tibia/fibula/ankle (S82), foot (S92), or unspecified (T02, T08, T10, T12). A clinical osteoporotic fracture was defined as a fracture of the radius/ulna, vertebrae, femur, hip, humerus, pelvis, or ribs.

### Risk Factors

The presence of risk factors was assessed by reviewing the computerized medical records for any evidence of risk factors before the start of each interval. Potential confounders that were determined at baseline were sex and a history of fracture. For a time-dependent analysis we considered age, a history of chronic diseases (rheumatoid arthritis, congestive heart failure, asthma, epilepsy, cerebrovascular disease, noninfectious enteritis and colitis, dementia, renal failure, diabetic retinopathy, hyperparathyroidism), as well as a prescription for thiazide diuretics, estrogen-containing hormone replacement therapy, statins, oral glucocorticoids, bisphosphonates, calcium, vitamin D, antipsychotics, antidepressants, antiarrhythmics, hypnotics/anxiolytics, anticonvulsants, opioids, and asthma medication in the previous 6 months as potential confounders.

### Statistical Analysis

Cox proportional hazards analysis was used. In the first analysis, we compared fracture risk in users of TZDs with that in control patients (without diabetes) and users of other AD medication. In this analysis, calculations were adjusted for all potential confounders that changed the hazard ratio (HR) >1 % in an age-/sex-adjusted analysis. Secondly, we studied the risk of osteoporotic fracture in current AD drug users versus controls, using the proxy indicator for severity of disease. To investigate whether a dose-response relationship was apparent, we stratified the risk of osteoporotic fracture in current TZD users by number of TZD prescriptions ever before. Wald tests were used to examine if there were significant differences between differently exposed groups. All data management and statistical analyzes were conducted using SAS 9.1/9.2 software (SAS Institute, Cary, NC).

## Results

Our study population included 180,049 diabetic patients and 490,147 population-based controls. Mean age at index date (first OAD prescription) was 62.6 years, and 47 % of all patients were female. Mean duration of follow-up after the index date was 5.3 years for diabetic patients and 6.2 years for controls. Further baseline characteristics are shown in Table 1. During the study period, 4.2 % of diabetic patients were prescribed a TZD at least once (Table 2). Of all diabetic patients, 12.8 % suffered from a fracture after the index date. Hip fractures (3.1 %) and radius/ulna fractures (2.2 %) were common.

**Table 1:** Baseline characteristics of diabetic patients and controls

Characteristic	Diabetic patients (%)	Controls (%)
	(*n* = 180,049)	(*n* = 490,147)
Mean duration of follow-up after index date, years	5.3	6.2
Sex female	84,665 (47.0)	232,373 (47.4)
Age at index date		
Mean	62.6	62.5
By category		
18–29	4,064 (2.3)	10,174 (2.1)
30–39	8,164 (4.5)	21,029 (4.3)
40–49	18,887 (10.5)	50,622 (10.3)
50–59	39,944 (22.2)	111,053 (22.7)
60–69	47,288 (26.3)	133,197 (27.2)
70–79	40,586 (22.5)	111,448 (22.7)
80+	21,116 (11.7)	52,624 (10.7)
History of comorbidity		
Fracture	30,631 (17.0)	80,127 (16.3)
Asthma	5,706 (3.2)	9,126 (1.9)
Rheumatoid arthritis	2,232 (1.2)	5,188 (1.1)
Cerebrovascular disease	15,084 (8.4)	22,155 (4.5)
Epilepsy	2,615 (1.5)	5,960 (1.2)
History of drug use		
Statins	24,014 (13.3)	24,649 (5.0)
Antiarrhythmics	1,517 (0.8)	2,563 (0.5)
Antidepressants	26,774 (14.9)	52,861 (10.8)
Antipsychotics	13,232 (7.3)	21,813 (4.5)
Anxiolytics/hypnotics	51,984 (28.9)	119,634 (24.4)
Anticonvulsants	6,078 (3.4)	12,410 (2.5)
Opioids	41,930 (23.3)	78,901 (16.1)
Oral glucocorticoids	26,645 (14.8)	54,170 (11.1)
Bisphosphonates	1,668 (0.9)	6,368 (1.3)

**Table 2:** Use of AD medication and patients with fractures during follow-up

Characteristic	Diabetic patients (%)	Controls (%)
	(*n* = 180,049)	(*n* = 490,147)
Use of AD medication any time during follow-up		
Biguanide	122,751 (68.2)	–
sulfonyluerum	137,326 (76.3)	–
TZD	7,603 (4.2)	–
Insulin	42,525 (23.6)	–
Other	17,865 (9.9)	–
Any fracture	22,978 (12.8)	66,401 (13.5)
Foot/ankle	3,180 (1.8)	8,003 (1.6)
Tibia/fibula	2,274 (1.3)	5,552 (1.1)
Hand/wrist	2,694 (1.5)	8,187 (1.7)
Osteoporotic	14,910 (8.3)	44,031 (9.0)
Hip	5,642 (3.1)	15,166 (3.1)
Vertebral	1,221 (0.7)	3,341 (0.7)
Radius/ulna	3,884 (2.2)	15,138 (3.1)
Humerus	2,926 (1.6)	7,121 (1.5)
Ribs	772 (0.4)	2,087 (0.4)

*TZD* thiazolidinedione, *AD* antidiabetic

Table 3 shows that current TZD users had an increased risk of fracture versus nondiabetic patients (HR = 1.29, 95 % CI 1.13–1.49) but no increased risk of fracture versus patients who were exposed to other AD drugs (HR = 1.11, 95 % CI 0.97–1.27). When compared with other AD drug users, the risk of fracture with current TZD use was, however, increased in women (HR = 1.30, 95 % CI 1.10–1.54) and risks of foot/ankle fractures (HR = 1.54, 95 % CI 1.17–2.04) and tibia/fibula fractures (HR = 1.70, 95 % CI 1.22–2.37) were significantly increased. No increased risk of hip fracture was apparent for current users of TZDs.

**Table 3:** Risk of fracture in current TZD users compared with nondiabetic patients and with other AD drug users, by type of fracture and sex

	Current TZD users versus nondiabetic patients			Current TZD users versus other AD drug users		
	Fracture, *n*	Age-/sex-adj. HR (95 % CI)	Fully adj. HR (95 % CI)	Fracture, *n*	Age-/sex-adj. HR (95 % CI)	Fully adj. HR (95 % CI)
No TZD use	66,401			21,202		
Current TZD use						
Any fracture	213	1.24 (1.09–1.42)	1.29 (1.13–1.49)^a^	213	1.03 (0.90–1.18)	1.11 (0.97–1.27)^h^
Foot/ankle	52	1.96 (1.49–2.58)	1.84 (1.38–2.44)^b^	52	1.50 (1.14–1.98)	1.54 (1.17–2.04)^i^
Tibia/fibula	36	2.18 (1.57–3.03)	2.14 (1.52–3.02)^c^	36	1.64 (1.17–2.28)	1.70 (1.22–2.37)^i^
Hand/wrist	22	0.83 (0.55–1.27)	0.82 (0.54–1.24)^d^	22	0.76 (0.50–1.16)	0.79 (0.52–1.20)^i^
Osteoporotic	116	1.21 (1.01–1.45)	1.28 (1.06–1.54)^a^	116	0.98 (0.81–1.18)	1.07 (0.89–1.29)^j^
Hip	19	0.80 (0.51–1.26)	0.89 (0.57–1.39)^e^	19	0.53 (0.34–0.84)	0.61 (0.39–0.96)^k^
Femur	2	–^n^		2	–^n^	
Vertebral	11	1.44 (0.80–2.61)	1.67 (0.92–3.03)^f^	11	1.00 (0.55–1.81)	1.12 (0.62–2.04)^e^
Radius/ulna	43	1.11 (0.82–1.50)	1.10 (0.81–1.48)^d^	43	1.23 (0.91–1.67)	1.27 (0.94–1.73)^i^
Humerus	28	1.64 (1.13–2.38)	1.60 (1.09–2.36)^a^	28	1.08 (0.75–1.58)	1.16 (0.80–1.69)^l^
Pelvis	3	–^n^		3	–^n^	
Ribs	11	1.85 (1.02–3.35)	1.87 (1.01–3.48)^g^	11	1.38(0.76–2.52)	1.47 (0.81–2.68)^m^
By sex						
Male^o^	74	0.99 (0.79–1.24)	1.06 (0.84–1.34)	74	0.79 (0.63–1.00)	0.86 (0.68–1.09)
Female^p^	139	1.43 (1.21–1.69)	1.46 (1.23–1.73)	139	1.22 (1.03–1.44)	1.30 (1.10–1.54)

*TZD* thiazolidinedione, *AD* antidiabetic, *adj* adjusted, *HR* hazard ratio, *CI* confidence interval

^a^Adjusted for age; gender; use of statins, bisphosphonates, antidepressants, or opioids in the previous 6 months; history of fracture; and diabetic retinopathy

^b^Adjusted for age; gender; use of statins, antidepressants, or opioids in the previous 6 months; history of fracture; and diabetic retinopathy

^c^Adjusted for age; gender; use of statins, antidepressants, anticonvulsants, or opioids in the previous 6 months; history of fracture; and diabetic retinopathy

^d^Adjusted for age, gender, use of antidepressants or opioids in the previous 6 months, and history of fracture

^e^Adjusted for age; gender; use of statins, bisphosphonates, antidepressants, or opioids in the previous 6 months; history of fracture; and cerebrovascular disease

^f^Adjusted for age; gender; use of statins, bisphosphonates, antidepressants, or opioids in the previous 6 months; history of fracture; diabetic retinopathy; and cerebrovascular disease

^g^Adjusted for age; gender; use of statins, bisphosphonates, antidepressants, hypnotics/anxiolytics, anticonvulsants, or opioids in the previous 6 months; history of fracture; diabetic retinopathy; and cerebrovascular disease

^h^Adjusted for age, gender, use of statins or antidepressants in the previous 6 months, history of fracture, and cerebrovascular disease

^i^Adjusted for age, gender, use of statins in the previous 6 months, and history of fracture

^j^Adjusted for age; gender; use of statins, antidepressants, or opioids in the previous 6 months; history of fracture; and cerebrovascular disease

^k^Adjusted for age; gender; use of statins, antipsychotics, antidepressants, or opioids in the previous 6 months; history of fracture; cerebrovascular disease; and dementia

^l^Adjusted for age; gender; use of statins, antipsychotics, or antidepressants in the previous 6 months; history of fracture; and cerebrovascular disease

^m^Adjusted for age, gender, use of statins or antidepressants in the previous 6 months, and history of fracture

^n^Number of cases too low to estimate HR

^o^Male TZD users versus male nonusers

^p^Female TZD users versus female nonusers

When we stratified current AD drug users by the proxy indicator of disease severity, we found that the risk of osteoporotic fracture was 1.3-fold increased for stages 3 and 4 compared with nondiabetic patients (Table 4). Patients who were currently using TZDs (stage 3 HR = 1.27, 95 % CI 1.06–1.52) had a similar risk of osteoporotic fracture as patients who were currently using insulin (stage 4 HR = 1.25, 95 % CI 1.20–1.31). In women only, we found a similar result using the proxy indicator of disease severity. The risk in stage 3 was slightly higher than that in stage 4, but this difference was not significant based on a Wald test (stage 1 fully adjusted HR = 1.11, 95 % CI 1.07–1.14; stage 2 HR = 0.98, 95 % CI 0.93–1.04; stage 3 HR = 1.34, 95 % CI 1.07–1.67; stage 4 HR = 1.17, 95 % CI 1.10–1.23). In men only, the risk in stage 3 was not significantly increased (stage 1 fully adjusted HR = 1.25, 95 % CI 1.20–1.31; stage 2 HR = 1.02, 95 % CI 0.94–1.10; stage 3 HR = 1.13, 95 % CI 0.82–1.57; stage 4 HR = 1.41, 95 % CI 1.31–1.52). In men, the risk in stage 3 was not significantly different from the risk in stage 4 either.

**Table 4:** Risk of osteoporotic fracture in current AD drug users compared with controls, by stage of disease

	Fracture, *n*	Age-/sex-adj. HR (95 % CI)	Fully adj. HR (95 % CI)^a^
No diabetes	44,031	1	1
Current AD drug use	13,989	1.21 (1.18–1.23)	1.14 (1.12–1.16)
By stage of disease			
Stage 1: current use of biguanide or sulfonyluerum	8,866	1.23 (1.20–1.26)^b,c^	1.15 (1.13–1.18)^b,c^
Stage 2: current use of biguanide and sulfonyluerum	2,222	1.01 (0.97–1.05)^d^	1.00 (0.96–1.04)^d,e^
Stage 3: current use of TZD^f^	116	1.20 (1.00–1.44)	1.27 (1.06–1.52)
Stage 4: current use of insulin^g^	2,419	1.37 (1.32–1.43)	1.25 (1.20–1.31)
Unclassified stages	366	1.14 (1.03–1.26)	1.11 (1.00–1.23)

*TZD* thiazolidinedione, *AD* antidiabetic, *adj* adjusted, *HR* hazard ratio, *CI* confidence interval

^a^Adjusted for age; gender; use of statins, antipsychotics, antidepressants, or opioids in the previous 6 months; history of fracture; diabetic retinopathy; congestive heart failure; and cerebrovascular disease

^b^Statistically significant difference (*p* < 0.05) between current AD users in stage 1 and current AD users in stage 2, based on Wald test

^c^Statistically significant difference (*p* < 0.05) between current AD users in stage 1 and current AD users in stage 4, based on Wald test

^d^Statistically significant difference (*p* < 0.05) between current AD users in stage 2 and current AD users in stage 4, based on Wald test

^e^Statistically significant difference (*p* < 0.05) between current AD users in stage 2 and current AD users in stage 3, based on Wald test

^f^Regardless of comedication

^g^Regardless of comedication and not exposed to TZDs

Stratification of current TZD users by the number of TZD prescriptions ever before showed a cumulative dose–response relationship (Table 5). Current TZD users with 30 or more TZD presciptions ever before had a threefold increased risk of osteoporotic fracture compared with other AD drug users (HR = 3.03, 95 % CI 2.03–4.52).

**Table 5:** Risk of osteoporotic fracture in current TZD users compared with other AD drug users, by number of TZD prescriptions

	Fracture, *n*	Age-/sex-adj. HR (95 % CI)	Fully adj. HR (95 % CI)^a^
No TZD use	13,873		
Current TZD use	116	0.98 (0.81–1.18)	1.07 (0.89–1.29)
By number of TZD prescriptions ever before			
1–4	27	0.80 (0.55–1.17)	0.87 (0.59–1.26)
5–8	17	0.70 (0.44–1.13)	0.76 (0.47–1.23)
9–14	20	0.80 (0.52–1.24)	0.88 (0.57–1.37)
15–29	28	1.05 (0.72–1.52)	1.16 (0.80–1.68)
≥30	24	2.70 (1.81–4.02)	3.03 (2.03–4.52)^b^

*TZD* thiazolidinedione, *AD* antidiabetic, *adj* adjusted, *HR* hazard ratio, *CI* confidence interval

^a^Adjusted for age; gender; use of statins, antidepressants, or opioids in the previous 6 months; history of fracture; and cerebrovascular disease

^b^Statistically significant difference (*p* < 0.05) between current TZD users with ≥30 TZD prescriptions ever before and all of the other categories, based on Wald test

## Discussion

This study showed a 1.3-fold increased risk of fracture in current TZD users versus nondiabetic patients. Overall, current TZD users had no significantly increased risk of fracture compared with users of other AD drugs. The risk of fracture with current TZD use was, however, increased in women, among prolonged users of TZDs, and risks of foot/ankle and tibia/fibula fractures were significantly increased.

Our finding of an increased risk of fracture in women using TZDs versus other AD drug users is in line with previous studies. In a meta-analysis from randomized controlled trials [10], the pooled data from the five trials that reported fracture risk by sex showed a significantly increased risk of fractures among women (OR = 2.23, 95 % CI 1.65–3.01) but not among men (OR = 1.00, 95 % CI 0.73–1.39). Another recent trial on rosiglitazone use presented a similar result: there was an increased fracture risk in women (RR = 1.82, 95 % CI 1.37–2.41) but not in men (RR = 1.23, 95 % CI 0.85–1.77) [27]. In observational studies, the use of TZDs has also been linked with an increase in fracture risk [12–14]. Habib et al. [12] found in their cohort study that TZD use was associated with a 1.6-fold increased risk of fracture in women but not in men. Conversely, Douglas et al. [13] reported an increased risk of fracture in both women and men who were exposed to TZDs. In a nested case–control study [11] an increased risk of fracture with TZD use was only apparent in the group with cumulative exposure of 15 TZD prescriptions or more (HR = 2.86, 95 % CI 1.57–5.22). This result is in line with our finding of a significantly increased risk of osteoporotic fracture for current TZD users in the stratum with highest cumulative exposure.

The association between TZD use and fracture risk may be explained by the drug itself but also by the underlying disease because both T2DM and the use of TZDs seem to be independently associated with an increased risk of adverse skeletal effects. There is substantial evidence from in vitro studies that activation of PPARγ, which is stimulated by TZDs, causes a predominant formation of adipocytes from mesenchymal stem cells, while the development of osteoblasts is suppressed [4, 5]. Moreover, BMD was significantly reduced at the lumbar spine and hip in women exposed to TZDs in two randomized controlled trials [8, 9].

In addition, epidemiological studies suggest that T2DM is an independent risk factor for adverse effects on the skeleton [15, 16]. Even though patients with T2DM have on average a higher BMI [28] as well as a higher BMD [16] compared with the general population, which would in theory protect them against fractures, previous studies have found an increased risk of fractures in T2DM [15]. This finding may be explained by different mechanisms. High blood glucose might have a direct toxic effect on bone cells [17, 29] and may cause a negative calcium balance by increased urinary calcium excretion [18] and reduced intestinal calcium absorption [30]. There is evidence that vitamin D metabolism is altered in diabetic patients [19, 31], which may lead to bone loss. Further, functional hypoparathyroidism may result in low bone turnover [32]. Complications of diabetes, such as neuropathy and angiopathy, may also contribute to an increased risk of fracture as well as disease severity in general [21–24].

To evaluate to what extent the association between TZD use and fracture risk is related to the drug or to the underlying disease, we created a proxy indicator of disease severity. We defined four different stages based on a prescription scenario that is often used in the treatment of T2DM. Patients who were currently using insulin (stage 4) had a significantly greater risk of osteoporotic fracture than patients in stages 1 and 2, which supports the hypothesis that the underlying disease is involved. If the drug itself were strongly related to fracture risk, we would expect a higher risk in patients who were currently exposed to TZDs (stage 3). However, patients in stage 3 had a similar risk of osteoporotic fracture as patients in stage 4. Therefore, we would argue that the association between TZD use and fracture is at least partially confounded by the underlying disease.

Strengths of our study include a reasonable sample size and duration of follow-up. Our source population included the total Danish population, and controls were therefore population-based. There was detailed longitudinal information on drug prescribing.

There are several limitations. Data on smoking and body mass index (BMI) were not available, and therefore, unmeasured confounding may have biased our findings. Patients with T2DM have on average a higher BMI than the general population [28], which is protective against fracture [33]. In our study, this may have led to underestimation of a true association between T2DM/TZD use and fracture. However, we did adjust our analyzes for the use of statins, which is probably correlated with high BMI. Using a proxy indicator of disease severity, we may have underestimated the role of the underlying T2DM as a confounder in the association between TZD use and fracture. We used current treatment with insulin as a proxy indicator for the highest level of disease severity (stage 4). The risk of fracture for patients in this stage may be biased by potential direct effects of insulin on bone. Although the role of insulin on fracture risk is still unclear, it has been suggested that higher insulin levels in T2DM may preserve and increase bone density and bone strength [30, 34]. If insulin were indeed protective against fractures, this may have resulted in underestimation of a true association between disease severity and fracture risk.

In conclusion, we found that patients who were currently using TZDs had a similar risk of osteoporotic fracture as patients who were currently using insulin. When observational studies assess the risk of fracture in patients using TZDs, the underlying T2DM should be taken into account.
